# Editorial: The management of hematologic malignancies in lower-income countries

**DOI:** 10.3389/fonc.2023.1218718

**Published:** 2023-05-22

**Authors:** Prasanth Ganesan, Jean El Cheikh, Alessandro Isidori, Sung-Hsin Kuo, Mustafa Saleh, Reena Nair

**Affiliations:** ^1^ Medical Oncology, Jawaharlal Institute of Postgraduate Medical Education and Research (JIPMER), Pondicherry, India; ^2^ Department of Hematology-Oncology, American University of Beirut Medical Center, Beirut, Lebanon; ^3^ Hematology and Stem Cell Transplant Center, Marche Norde Hospital, Pesaro, Italy; ^4^ Graduate Institute of Oncology, College of Medicine, National Taiwan University, Taipei, Taiwan; ^5^ Clinical Haematology Oncology and Hematopoietic Cell Transplantation (HCT), Tata Medical Centre, Kolkata, India

**Keywords:** lower and middle income countries, hematological malignancies, allogenic stem cell transplantation, infectious complication, survival

Advances in modern treatments, interdisciplinary research, and supportive care have led to a decline in cancer mortality while improving remission rates, overall survival, and quality of life (1). Unfortunately, this progress is not accessible to all, as effective care is only available in high-income countries, leaving behind most of the world’s patients in lower-income countries (LMICs) (2). This disparity in cancer patient care and outcomes has widened over time, underscoring the importance of prevention, treatment, and palliative care in LMICs (3).

Managing hematologic malignancies in LMICs presents unique challenges, including delayed presentation, missing the diagnosis, limited access to medication, financial or economic burden, and patient adherence to therapy (4). Furthermore, infectious complications remain a vexing problem, contributing to increased acute morbidity and mortality, even in centers that offer state-of-the-art care. This series of articles on hematologic malignancies in LMICs gives insights into the problems that persist in managing these diseases. Despite covering a range of diagnoses, all the papers highlight the critical need for effective strategies to address the challenges of managing hematologic malignancies in LMICs.

In their article, Salama et al. from Egypt discuss the challenges in managing mixed-phenotype acute leukemia (MPAL) in LMICs. MPAL is a relatively rarer subtype of acute leukemias, and there are limited data, especially on children from LMICs (5, 6). With little data available on this rare subtype of acute leukemias, the authors report on a series of 42 children treated for MPAL, achieving a 5-year event-free survival (EFS) of 56% and overall survival (OS) of 61%. These results are comparable to data from two pediatric MPAL series in India, highlighting the challenges in managing high-risk pediatric leukemias in resource-limited settings (5, 6). In one of the largest reports of pediatric MPALs from LMICs, the authors highlight the difficulties of treating high-risk pediatric leukemia in resource-limited settings. This includes the non-availability of allogeneic transplants (only five patients in this series) for patients with residual disease, high therapy-related mortality with acute myeloid leukemia (AML) regimens (17%), and non-availability of targeted agents (e.g., anti-Flt-3, bispecific monoclonals, antibody–drug conjugates). The authors highlight that minimal residual disease (MRD) use was high, which is commendable. Still, very few patients received allogeneic transplants based on MRD positivity; this points to an opportunity for improved management strategies. While no prospective trials exist, the data from this study reiterate the critical need for effective management strategies for hematologic malignancies in LMICs, particularly in the case of rare subtypes such as MPAL.

The second article of this series, authored by Hammad et al. from Egypt, has a specific focus on allogeneic-matched sibling donor (MSD) hematopoietic stem cell (HSCT) transplants in pediatric patients diagnosed with acute lymphoblastic leukemia (ALL). The study examines the outcomes of 67 patients who received allogeneic HSCT, most of which was conducted for patients with Philadelphia chromosome-positive (Ph+) ALL and those who failed to achieve remission during induction therapy. The most common conditioning regimen was total body irradiation (TBI) (79%), along with cyclosporine and methotrexate as the graft-versus-host disease (GvHD) prophylaxis. The study reports grade III-IV GvHD rates of 15% and non-relapse mortality of 15%, figures comparable to other series of MSD HSCT from developing countries. The findings suggest a 5-year EFS rate of 49% (56% in CR1 and 32% in CR2) and an OS rate of 56%. Although current practice in pediatric (Ph+) ALL does not typically involve offering CR1 allotransplant, the authors in this series have used it as an institutional practice, which resulted in an impressive EFS of 89%. However, patients with induction failure had poorer outcomes, with an EFS of 29%, even after achieving MRD <0.01% before transplant. The treatment of high-risk and relapsed leukemia remains a significant challenge in LMICs. Despite selected centers performing allogeneic HSCTs, the results are generally poor in cases of refractory ALL. One potential solution to this problem is increasing access to agents such as blinatumomab or CAR-T cells, which have shown recent promise in expanding the depth and duration of response.

One of the vexing problems in LMICs, especially when using intensive chemotherapy regimens, is the risk of infection and sepsis-related mortality. The above articles underscore the challenge of infection management in the context of hematologic malignancies. Clinicians practicing in these regions constantly search for solutions to mitigate this issue. Fear of infections limits the use of fludarabine-based regimens in many centers. In a well-conducted phase II study from the Republic of Korea, Jeon et al. used pegfilgrastim with a rituximab–fludarabine–cyclophosphamide (R-FC) regimen in chronic lymphoid leukemia (CLL). They reduced treatment-related mortality from 10% to 6%. With the availability of biosimilar versions of pegfilgrastim worldwide, the cost of pegfilgrastim has decreased significantly and increased access (7). Appropriate use of this agent can, thus, reduce the risk of febrile neutropenia (from 60-80% to 10-15% in this study) with R-FC regimens, thus saving on the costs of supportive care and saving lives.

This Research Topic focuses on tailoring contemporary treatments for hematological cancers in resource-limited conditions. The use of peg GCSF in R-FC is a prime example of a straightforward and practical solution as a supportive care treatment that allows for the safe administration of intense chemotherapy. While some of the results may seem inferior to those reported from affluent nations, it should be noted that most of these are real-world patients and ones not selected for clinical trials.

## Author contributions

PG: Writing the Manuscript, Revision and Approving the final version. JE: Writing the Manuscript, Revision and Approving the final version. AI: Writing the Manuscript, Revision and Approving the final version. S-HK: Writing the Manuscript, Revision and Approving the final version. MS: Writing the Manuscript, Revision and Approving the final version. RN: Writing the Manuscript, Revision and Approving the final version.
